# Comparison of the marginal and internal fit of PMMA interim crowns printed with different layer thicknesses in 3D‐printing technique

**DOI:** 10.1002/cre2.758

**Published:** 2023-06-29

**Authors:** Mahya Hasanzade, Negin Yaghoobi, Parsa Nematollahi, Rezvaneh Ghazanfari

**Affiliations:** ^1^ Department of Prosthodontics, School of Dentistry, International Campus Tehran University of Medical Sciences Tehran Iran; ^2^ Department of Prosthodontics, School of Dentistry Tehran University of Medical Sciences Tehran Iran; ^3^ School of Dentistry, International Campus Tehran University of Medical Sciences Tehran Iran

**Keywords:** 3D‐printing, interim dental prostheses, internal adaptation, marginal adaptation

## Abstract

**Objective:**

The aim of this in vitro study was to compare the effect of printing layer thickness on the marginal and internal fit of interim crowns.

**Material and methods:**

A maxillary first molar model was prepared for ceramic restoration. Thirty‐six crowns were printed with three different layer thicknesses using a digital light processing‐based three‐dimensional printer (25, 50, and 100 µm [LT 25, LT 50, and LT 100]). The marginal and internal gaps of the crowns were measured with replica technique. An analysis of variance was conducted to determine if there were significant differences between the groups (*ɑ* = .05).

**Results:**

The marginal gap of LT 100 group was significantly higher than that LT 25 (*p* = .002) and LT 50 groups (*p* ≤ .001). The LT 25 group has significantly larger axial gaps than LT 50 group (*p* = .013); however, there were no statistically significant differences between other groups. The LT 50 group showed the smallest axio‐occlusal gap. The mean occlusal gap differed significantly by printing layer thickness (*p* ≤ .001), with the largest gap occurring for LT 100.

**Conclusions:**

Provisional crowns printed with 50 µm layer thickness provided the best marginal and internal fit.

**Clinical significance:**

It is recommended that provisional crowns be printed with a 50 µm layer thickness to ensure optimal marginal and internal fit.

## 1 INTRODUCTION

Interim restorations provide esthetic, function, tooth positional stability, and protect vital prepared teeth and periodontal tissue (Aldahian et al., 2021; Alharbi et al., 2016; Gratton & Aquilino, 2004; Pituru et al., 2020). How a provisional crown fit is closely related to how it is fabricated. The fabrication method can take either a direct or indirect route, depending on the method of choice (Burns et al., 2003; Regish et al., 2011). Typically, an immediate provisional restoration is applied immediately after preparation as part of the direct restoration method, using Bis‐acrylics (categorized into two subgroups, UDMA and BIS‐GMA) as the material (Lee et al., 2022; Tom et al., 2016). This method has the advantages of being convenient, straightforward, relatively simple, and low cost of production (Astudillo‐Rubio et al., 2018; Regish et al., 2011). However, several disadvantages are associated with this method, including shrinkage during polymerization, marginal discrepancy, and heat generation (Regish et al., 2011; Tom et al., 2016).

A new indirect method that uses computer‐aided design/computer‐aided manufacturing technologies (CAD/CAM) as an alternative to the conventional direct method could potentially result in a faster laboratory procedure, increasing productivity, and providing consistently high‐quality products, as opposed to the conventional direct method (Al‐Humood et al., 2023; Fasbinder, 2010; Jain et al., 2022). In CAD/CAM dental systems, methods used in fabricating dental restorations can be categorized as subtractive manufacturing (SM) or additive manufacturing (AM) (Cortina et al., 2018; Uzun, 2008).

Although the SM technique is an accurate method of producing restorations, the high cost of materials and equipment has led to the use of AM (three‐dimensional [3D] printing) technology, which is believed to be able to produce a more accurate prosthesis without requiring more resources (Sulaiman, 2020). The 3D printing process involves adding powders and liquids layer by layer to build up the product. Layers are polymerized and merged during photopolymerization (Jawahar & Maragathavalli, 2019; Kessler et al., 2020). It has been found that there is significant shrinkage during polymerization, and this phenomenon may be because of the distance between the atoms of the monomers with low molecular weight is reduced during the process of polymerization, which in theory is caused by the reduction of the chemical distance between the atoms (Kotz et al., 2019).

Different AM techniques are frequently used for biomedical applications including digital light processing (DLP), inkjet, stereolithography, selective laser sintering, and fused deposition modeling (Brambilla et al., 2021). DLP method uses a light projector to polymerize photosensitive resin. High precision, faster fabrication time, and high printing resolution are some advantages of this technology. An acrylic or epoxy‐based light‐curing resin is used in DLP to produce prostheses through the process of photopolymerization. The light is irradiated into a tank containing an acrylic or epoxy‐based light‐curing resin, which is exposed to ultraviolet light (Brambilla et al., 2021; Quan et al., 2020).

An important factor of long‐term clinical success of restorations is to ensure that it is internally and marginally adapted (Blatz, 2002). Poor marginal fit causes microleakage, plaque accumulation, and inflammation of the gingiva (Contrepois et al., 2013; Nawafleh et al., 2013). In the case of interim crowns made from 3D‐printed materials, certain factors might influence their marginal accuracy; including speed of printing, intensity, built angle, number of layers, hardware, interlayer shrinkage, amount of supportive material, post‐fabrication process, printing time, and the thickness of the layers (Bona et al., 2021). The thickness of 3D printing layers is a controllable parameter which affects the accuracy of interim crowns (Çakmak et al., 2022). In this regard, proper adjustment of layer thickness is crucial in achieving optimal clinical results (Çakmak et al., 2021). Moreover, it has been shown that fabrication thickness can influence printing time (Çakmak et al., 2021; Sabbah et al., 2021). The thickness of layers can be set between 20 and 150 µm in photopolymerization‐based 3D printing (Revilla‐León & Özcan, 2019).

Different methods have been explained for measuring internal and marginal gaps including triple scan protocol, direct visualization with laser videography, microcomputed tomography (micro‐CT), profile projection, light microscopy, stereomicroscopy, and reference point matching (Onlay, 2018). It is thought that the replica technique is the most popular method for two‐dimensional clinical diagnosis, due to its noninvasive, simple, and inexpensive nature with a high degree of precision and repeatability (Park et al., 2016; Svanborg, 2020). Based on research comparing the reliabilities of different conventional and digital methods for evaluating the marginal fit of restorations, no significant differences were found (Hasanzade et al., 2020; Mai et al., 2020).

Although previous studies evaluated trueness and hardness of 3D‐printed interim restorations of varying thicknesses (Çakmak et al., 2021, 2022), limited research has been conducted on the marginal and internal fit of them (Gad & Fouda, 2023; Yang et al., 2022). Therefore, the purpose of this study was to investigate the internal and marginal fit of provisional crowns made using DLP‐based 3D printing at different layer thicknesses (25, 50, and 100 µm). Based on the null hypothesis, the thickness of the printing layer would not affect the marginal or internal fit of interim crowns.

## 2 MATERIALS AND METHODS

**Figure 1:**
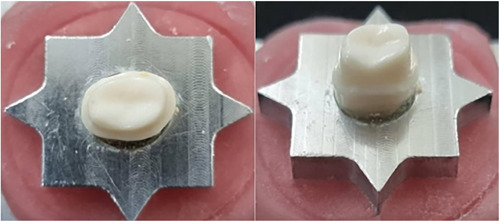
Maxillary right first molar preparation.

Using a typodont tooth (Nissin Dental Products), the first molar of the maxilla was fixed in a star‐shaped pattern base and then scanned by a laboratory digital scanner (Shining 3D ex pro). The model was prepared for all‐ceramic full‐coverage restoration including a 1.5 mm reduction of lingual cusps (functional) and 1 mm reduction of buccal cusps (nonfunctional) and a circumferential deep chamfer margin configuration with a 1 mm wide was carried out by cylindrical round‐end coarse diamond bur (#806 314 199 534, Ø18, Jota). Finishing was completed with a round‐end fine diamond bur (#806 314 199 504, Ø18, Jota) (Figure 1).

**Figure 2:**
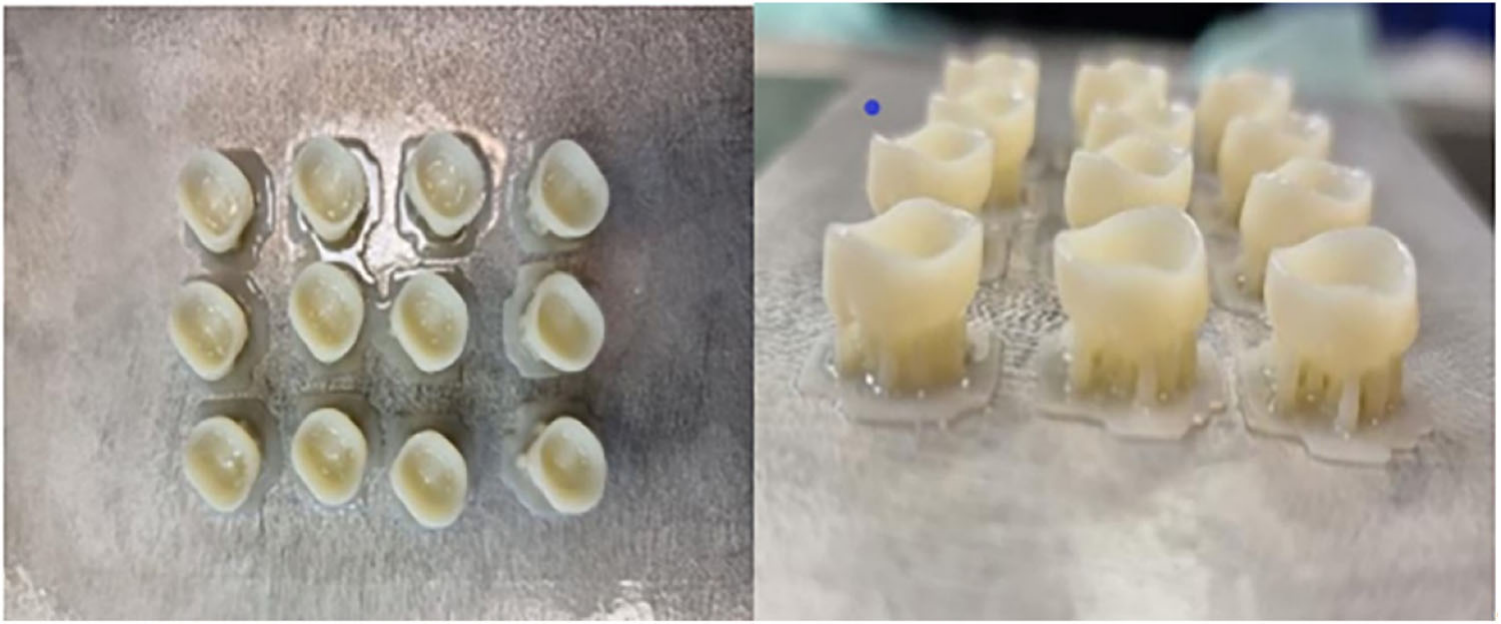
Three‐dimensional‐printed interim crowns (left: 25 μm crowns; middle: 50 μm crowns; right: 100 μm crowns).

Next, the prepared tooth was scanned by the same digital lab scanner. The crown was designed with an 80 µm cement gap (Dauti et al., 2020; Özçelik et al., 2018) according to the initial scan (unprepared tooth model) and the secondary scan (prepared tooth model). The design was converted into STL format and the data was transferred to the DLP‐based 3D printer (Digident plus). The interim crowns were made from acrylic‐based urethane methacrylate photopolymer resin (PowerDent Temp resin, Protech) with the following setting in three different groups (*n* = 12 for each group) at three thicknesses including 25 µm (59 min and 10 s printing time), 50 µm (33 min and 19 s printing time), and 100 µm (22 min and 35 s printing time), and the 45° build orientation was selected according to previous studies (Alkhateeb et al., 2023; Yang et al., 2022) (Figure 2).

This study utilized a printer with the following characteristics, as stated by the manufacturer. A UV light‐emitting diode with a wavelength of 405 nm, a projector resolution of 1280 × 800, a print size of 90 × 56 × 130 mm, a product size of 450 × 410 × 900 mm with an XY resolution of 25–100 µm and a *z*‐resolution of 1 µm. The fabricated crowns were washed using an ultrasonic bath (Digital ultrasonic cleaner, Skymen) filled with 98% isopropyl alcohol solution (Shera Ultra‐P; Technologie GmbH & Co.) for 180 s to clean unpolymerized and excessive resins. Then, for post curing, they were positioned in light cure unit (light zone, Denstar) for 10 min at 60°C (temperature was adjustable but is reported that 40–80°C improves mechanical and loading properties) (Tian et al., 2021).

**Figure 3:**
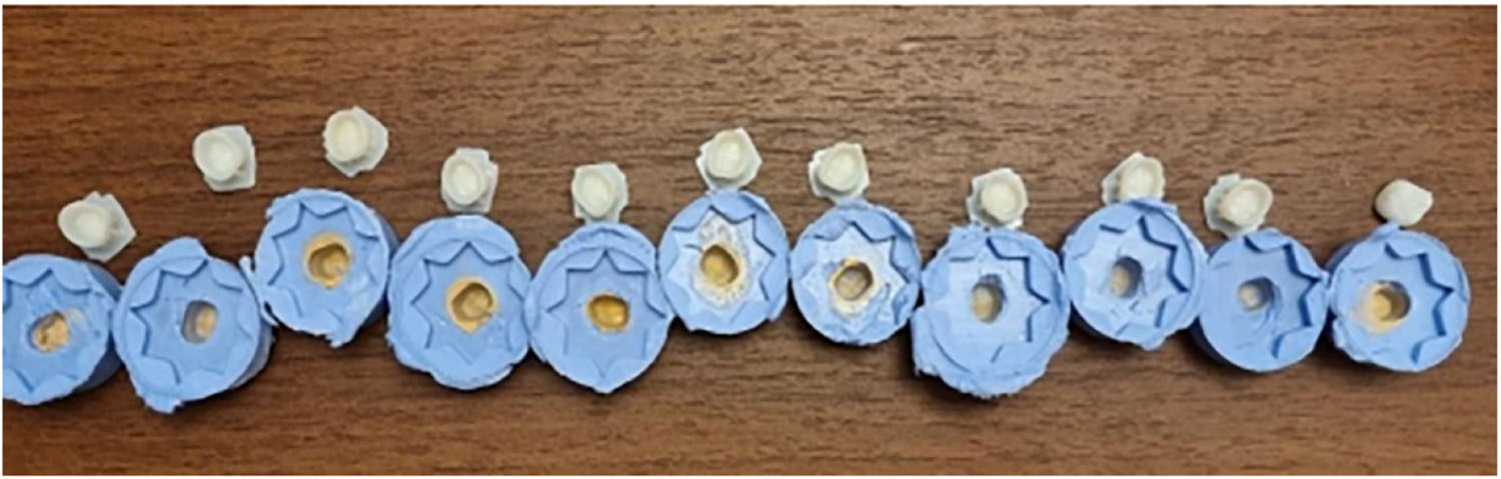
The replica technique and the direction of the section was placed at the corner of starlike pattern.

To determine the fit of the interim crowns internally and marginally, a replica technique was employed. First, a light body silicone was used to fill the interim crown (CharmFlex‐Light XLV), then the crown was placed on the prepared tooth model to be set by steady manual pressure to resemble the clinical situation (Nesse et al., 2015). After that, the interim crown was removed from the prepared tooth model while models were left with a thin layer of light body silicone. A special tray was filled with medium body silicone (CharmFlex‐Heavy) and placed on the model with light body silicon on it (Figure 3). It was necessary to cut each replica twice: once in the buccolingual direction and once in the mesiodistal direction, to divide each replica silicone into four pieces. For the standardization position of the cut, the direction of the section was placed at the corner of the starlike pattern and extended to the mentioned side.

**Figure 4:**
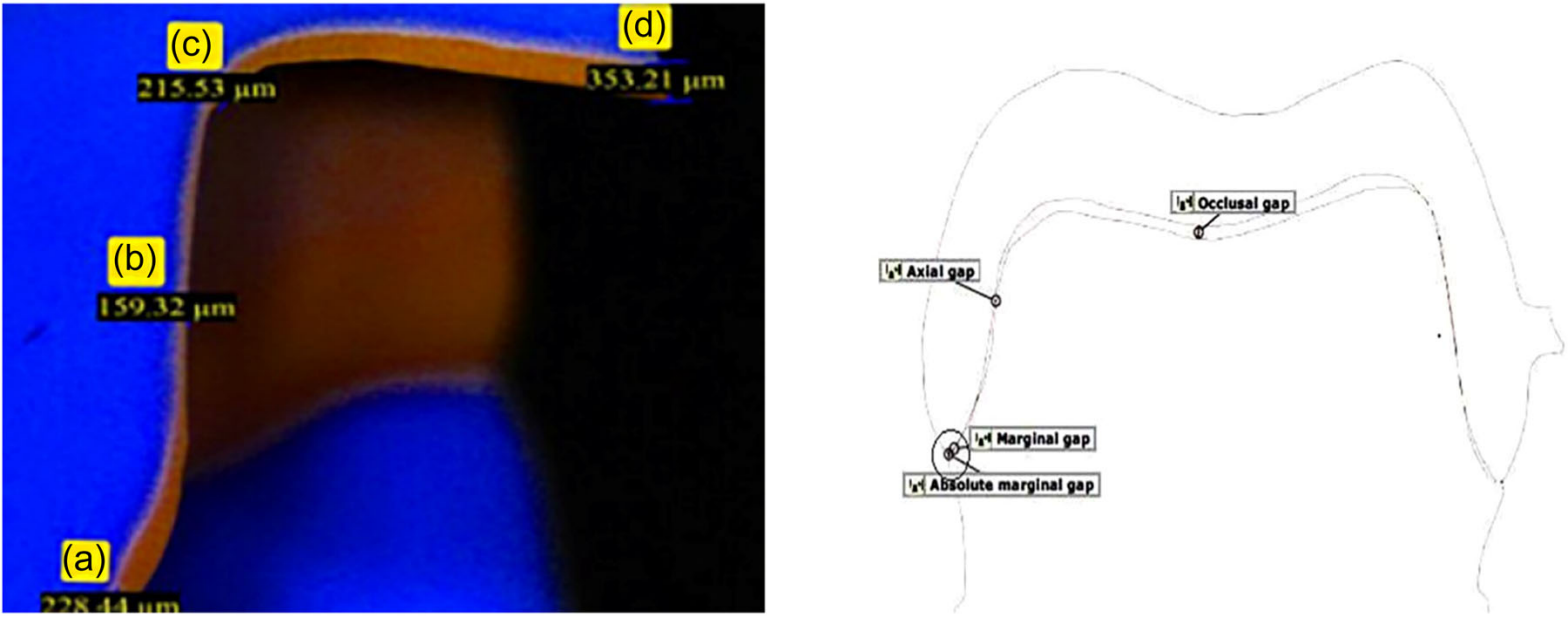
Assessing discrepancies by a stereomicroscope. (a) Marginal gap. (b) Axial gap. (c) Axio‐occlusal gap. (d) Occlusal gap.

Overall, four measurements were made for each one of the regions. The perpendicular measurement from the internal surface of crowns to prepared teeth at margin was considered as marginal gap. Internal gap was defined as the distance between the inner surface of the restoration and the prepared tooth's axial wall at mid‐axial, occlusal, and axio‐occlusal locations (Çakmak et al., 2022; Holmes et al., 1989). Each area was assessed by a blinded technician using a stereomicroscope (Olympus Bx 60; Olympus Optical) at ×12.5 magnification (Figure 4).

All of the analyses were done using Statistical Package for the Social Sciences (IBM Corp. Version 22.0). The normality of data for each dependent variable in each group was analyzed using Shapiro–Wilk test. To evaluate the marginal and internal gaps between the three groups, one‐way analysis of variance was used. After evaluating the homogeneity of variances (Leven's test) the multiple comparisons were done by Tukey honest significance test posthoc. The *p*‐value was considered <0.05 in all analyses.

## 3 RESULTS

Descriptive results including the mean and SD of the marginal and internal gaps are presented for each group in Table 1. In terms of marginal or internal gaps, significant differences were found among the three groups (*p* < .05). The marginal gap of LT 100 was significantly larger than LT 25 (*p* = .002) and LT 50 interim crowns (*p* ≤ .001). Additionally, the marginal gap for LT 25 was significantly greater than for LT 50 (*p* ≤ .001). In axial area, the mean gap in LT 25 was significantly higher than LT 50 (*p* = .013), whereas LT 100 was not significantly different from LT 50 in the axial gap (*p* > .05). Internal discrepancy at the midpoint of the axio‐occlusal walls was significantly greater in LT 25 and LT 100 in comparison to LT 50 (*p* ≤ .001). However, there were no statistically significant differences among LT 25 and LT 100. The occlusal gap demonstrated a remarkable difference among the three groups (*p* ≤ .001). The highest misfit value was for LT 100 (697.20 ± 82.92 µm), LT 50 (420.45 ± 92.41 µm), and LT 25 (282.83 ± 53.73 µm), respectively (Table 2). The total printing time was obtained and it is found that the crowns fabricated with 25 µm‐layer thickness had the longest printing time (59 min and 10 s) followed by 50 µm‐layer thickness (33 min and 19 s) and 100 µm‐layer thickness (22 min and 35 s).

**Table 1:** Marginal compared with internal discrepancies in 3D‐printed interim crowns.

Layer thickness (µm)	Average ±SD (µm)	Min (µm)	Max (µm)	Number
Marginal				
25	206.50 ± 66.22	159.00	240.00	12
50	159.93 ± 11.74	134.50	182.00	12
100	232.39 ± 14.07	205.75	254.00	12
Axial				
25	174.29 ± 30.70	136.00	222.25	12
50	147.62 ± 17.57	115.50	168.50	12
100	163.39 ± 12.51	145.50	187.00	12
Axio‐occlusal				
25	222.89 ± 30.73	160.50	275.00	12
50	174.33 ± 21.75	138.25	204.00	12
100	225.54 ± 14.35	194.25	244.50	12
Occlusal				
25	282.83 ± 53.73	205.00	353.00	12
50	420.45 ± 92.41	302.00	574.00	12
100	697.20 ± 82.92	268.00	770.00	12

Abbreviation: 3D, three‐dimensional.

**Table 2:** The intergroup comparisons of the marginal and internal gaps.

Dependent variable	(*I*) thickness of print (µm)	(*J*) thickness of print (µm)	Mean difference (*I* − *J*)	SE	Sig.
Marginal	25	50	46.56	7.05	0.000
		100	−25.89	7.05	0.002
	50	25	−46.56	7.05	0.000
		100	−72.45	7.05	0.000
	100	25	25.89	7.05	0.002
		50	72.45	7.05	0.000
Axial	25	50	26.66	8.84	0.013
		100	10.89	8.84	0.443
	50	25	−26.66	8.84	0.013
		100	−15.77	8.84	0.191
	100	25	−10.89	8.84	0.443
		50	15.77	8.84	0.191
Axio‐occlusal	25	50	48.56	9.49	0.000
		100	−2.64	9.49	0.958
	50	25	−48.56	9.49	0.000
		100	−51.20	9.49	0.000
	100	25	2.64	9.49	0.958
		50	51.20	9.49	0.000
Occlusal	25	50	−137.62	31.88	0.000
		100	−414.37	31.88	0.000
	50	25	137.62	31.88	0.000
		100	276.75	31.88	0.000
	100	25	414.37	31.88	0.000
		50	276.75	31.88	0.000

*Note*: The intergroup comparisons of the marginal and internal gaps in samples were 3D printed with different layer thickness, using one‐way ANOVA and Tukey's HSD, *p* < .05.

Abbreviations: 3D, three‐dimensional; ANOVA, analysis of variance; HSD, honest significance test; Sig., Significance.

## 4 DISCUSSION

There were significant differences in marginal and internal gaps among interim crowns fabricated with different thicknesses of printing layers that rendered the null hypothesis invalid. As a result of the study, it was found that the overall ranges of marginal, axial, axio‐occlusal, and occlusal gaps were 134–254, 115–222, 138–275, and 205–770 µm, respectively. Yao et al. (2014), Ryu et al. (2020), and Beuer et al. (2009) reported the range of marginal gap in interim crowns 150–280, 58–113, and 100–150 µm, respectively. In addition, Peng et al. (2020) reported a mean of 240 µm of the marginal gap in interim crowns made from PMMA fabricated by 3D printing, which was comparable to the range found in the present study. However, Chou et al. (2021) reported the range of internal gaps in permanent crowns fabricated by 3D printing was 92.15–120.20 µm, which was lower than the range obtained in this study. The main causes of difference could be using other 3D printing techniques, different devices, various factors such as built angle, type of supporting structure, types of materials used for crown fabrication, tooth model, variety in design, different finish lines, the amount of cement gap, and measurement methods.

In prior studies, the layer thickness of 3D‐printed custom trays and dental dies has been found to affect their mechanical properties (Liu et al., 2021; Sabbah et al., 2021). As a result of the study's evaluation of the bond, flexural, and tensile strength of custom PLA trays, as well as their dimensional accuracy, a medium‐layer thickness was found to provide the best mechanical behavior for all groups of specimens (Liu et al., 2021). Likewise, it was also investigated whether dies printed with different thicknesses of printing layers would have improved the surface roughness, repeatability, and dimensional stability of the dies. The researchers found that changing the layer thickness had no significant effect on surface roughness or repeatability (Sabbah et al., 2021).

Yang et al. (2022) evaluated the effect of build orientations and layer thickness on marginal fit and absolute marginal discrepancy of 3D‐printed three‐unit fixed partial denture (FPD) and they reported marginal fit of restorations was not affected by different printing thickness significantly. They calculated marginal fit of interim restorations printed with two different thickness (50 and 100 µm) in implant abutments using micro‐CT scanning technique. Limitation in range of printing thickness, different method of evaluating marginal fit, and differences in type of restorations (FPD or single crown in implant or teeth abutments) are possible explanation for different results (Yang et al., 2022).

Another study investigated the effect of printing layer thickness on flexural strength and surface hardness of bar‐shaped specimens. They reported that decreasing printing layer thickness enhanced flexural strength and surface hardness of specimens significantly. They mentioned that increase in light intensity when it passes through narrower bulk of material causes appropriate curing of layer (Perea‐Lowery et al., 2021).

Based om current study, all surfaces except the occlusal surface showed significantly lower discrepancies in the 50 µm thickness than in the other groups. Furthermore, the group with 100 μm of layers exhibited significantly greater marginal and occlusal gaps than the other two groups. Based on the results of this study one can conclude that added number of layers may lead to an increased cumulative error that reduces accuracy. Consequently, in 100 µm‐layer thickness, even though the cumulative error is decreased because of fewer layers, higher accuracy and fit are not possible because of thicker scanning and layer extrusion, which may limit appropriate curing (Zhang et al., 2019). Therefore, 50 µm‐layer thickness is the choice considering both layer thickness and cumulative error; the least gap level (best fit) was observed in the marginal and internal areas, except the occlusal surface. It should be noted that, unlike other examined areas, the occlusal gap was the lowest in 25 µm‐layer thickness. As the 3D printing was done from occlusal to gingival in this study, 25 µm‐layer thickness showed the lowest gap in this area. Çakmak et al. (2022) evaluated trueness of 3D‐printed FPDs printed with different printing thickness. In terms of intaglio and overall trueness, the 20 and 50 µm thickness groups did not show any difference in trueness, but they did show higher trueness than the 100 µm thickness group. An assessment of the marginal adaptation of provisional printed crowns with varying layers thickness was conducted in another study (Çakmak et al., 2021). As a result, the marginal quality of crowns with 50 µm layers is higher than those with a 20 and 100 µm thickness, and the difference between 20 and 100 µm crowns is nonsignificant (*p* = .34). The findings of the present work, similar to previous studies (Çakmak et al., 2021; Sabbah et al., 2021) showed that the crowns fabricated with 25 µm‐layer thickness had the longest printing time because of a higher number of layers (there is an inverse relation between the number of layers and layer thickness).

There were limitations to the present study, including the use of the replica technique to measure the internal and marginal fit, as it limited the number of evaluating points and areas compared to 3D methods; moreover, this method can be technique‐sensitive and be dependents to the examiner, dimensional stability of the intermedium material, and level of magnification. In addition, the printed crowns were seated on the prepared teeth with finger pressure, which is above the recommended seating pressure. Finally, for future studies, it is recommended to use triple‐scan or micro‐CT technique for measuring the marginal and internal gap of interim crowns. Furthermore, other properties of interim restorations such as color stability or hardness in different printing layer thickness can be evaluated in future studies.

## 5 CONCLUSION

Within the limitations of current study, it is important to consider the impact of 3D printing layer thickness on marginal and internal fit when designing a product, and 50 µm layer thickness had the best fit in all areas, except for the occlusal area where 25 µm layer thickness was the most suitable.

## AUTHOR CONTRIBUTIONS

Design of the article: Maya Hasanzade, Rezvaneh Ghazanfari. Draft of the manuscript: Maya Hasanzade, Negin Yaghoobi. Data Acquisition and analysis: Pars Nematollahi, Rezvaneh Ghazanfari, Maya Hasanzade. Critically revised the manuscript: Maya Hasanzade, Negin Yaghoobi, Rezvaneh Ghazanfari.

## CONFLICT OF INTEREST STATEMENT

The authors declare no conflicts of interest.

## Data Availability

Data available on request from the authors.
